# *Cordyceps militaris* Grown on Germinated Soybean Induces G2/M Cell Cycle Arrest through Downregulation of Cyclin B1 and Cdc25c in Human Colon Cancer HT-29 Cells

**DOI:** 10.1155/2012/249217

**Published:** 2012-03-12

**Authors:** Mohammad Lalmoddin Mollah, Dong Ki Park, Hye-Jin Park

**Affiliations:** Department of Bioscience and Biotechnology, Konkuk University, 1 Hwayang-dong, Kwangjin-gu, Seoul 143-701, Republic of Korea

## Abstract

*Cordyceps militaris* (CM) is an insect-borne fungus that has been used in traditional Chinese medicine because of its wide range of pharmacological activities. In this paper, we studied CM grown on germinated soybean (GSC) and investigated the possible mechanisms underlying antiproliferative effect of GSC on HT-29 human colon cancer cells. In comparison with CM extracts and germinated soybean (GS) BuOH extracts, BuOH extracts of GSC showed remarkable inhibitory and antiproliferative effects on HT-29 colon cancer cells. After GSC treatment, HT-29 cells became smaller and irregular in shape. High G2/M phase cell populations were observed in the GSC-treated group. The levels of cyclin B1 and Cdc25 in the GSC-treated group were lower than those in the control group. These findings suggest that GSC BuOH extracts might act as an effective anti-proliferative agent by inducing G2/M cell cycle arrest in colon cancer cells.

## 1. Introduction

Colon cancer is a serious public health problem in the Western world [1]. According to the American Cancer Society, colon cancer is the second leading cause of cancer-related deaths in the United States (US). Patient survival is related to the tumor stage. For example, stage I colorectal cancer, in which the carcinoma remains localized in the submucosa of the colon epithelium, has an overall 5-year survival rate of over 90% while the 5-year survival rate for stage IV cancer is less than 10%. The dysregulation of cell cycle found in some cancers. A large number of cells show modulated expression of the molecules responsible for apoptosis and cell cycle arrest [2], including cyclins and cyclin-dependent protein kinases (CDKs).

Current therapies for cancer are largely based on cancer-specific surgery, hormone therapy, radiotherapy, chemotherapy, and treatment with anticancer drugs. While advances continue to be made in the development of effective strategies for treating colorectal cancer, chemotherapy is often limited by the severe adverse effects and dose-limiting toxicity of the drugs. Therefore, it is extremely essential to identify and screen compounds from natural products that can effectively treat cancer with no adverse effects.

A large number of medicinal mushrooms have been proven to possess anticancer activities [3–6]. One such mushroom, *Cordyceps militaris* (CM) has been used in traditional Chinese medicine. CM extracts have been reported to exert a wide range of pharmacological activities, including immunomodulatory, anti-inflammatory, and antitumor activities [7–10]. Kim et al. [11] and Park et al. [7] reported that CM has potent cytotoxic effect against cancer cells and exerts immunostimulatory effects. Jin et al. reported that aqueous CM extracts induced apoptosis in human breast cancer MDA-MB231 cells by activating caspases and inactivating Akt [12].

Despite the clinical and pharmacological importance of CM, naturally occurring CM is not easily available in large quantities because of its high cost of production. Therefore, we developed novel methods for cultivating CM. We successfully cultivated CM on germinated soybeans (GS), which contain a large number of nutrients and active biological compounds such as isoflavones [13, 14]. In our previous study, we demonstrated that CM cultivated on GS (GSC) shows better pharmacological activity than naturally occurring CM [10, 15]. However, the activity of GSC against colon cancer carcinogenesis has not been clearly stated. In this study, we investigated the effect of GSC on HT-29 colon cancer cells and determined the molecular mechanism underlying this effect. 

## 2. Materials and Methods

### 2.1. Reagents and Chemicals

GSC, CM, and GS (Kucari 0903) were obtained from The Cell Activation Research Institute, Seoul, Republic of Korea. RPMI 1640 medium, fetal bovine serum (FBS), penicillin-streptomycin, and trypsin-EDTA were obtained from Gibco BRL (Grand Island, NY, USA). Propidium iodide (PI), NP-40, and RNase A were obtained from Sigma Chemical Co. (St. Louis, MO, USA). Anti-Cdc25c (Cell Signaling Technology Inc., Danvers, MA), anticyclin B1 (Cell Signaling Technology Inc., Danvers, MA), anti-Bcl2 (Cell Signaling Technology Inc., Danvers, MA), anticaspase-9 (Cell Signaling Technology Inc., Danvers, MA), anti-*β*-actin, horseradish peroxidase- (HRP-) conjugated anti-rabbit, and anti-mouse IgG antibodies (Santa Cruz Biotechnology, Santa Cruz, CA) were obtained from the respective suppliers. The chemiluminescence detection kits were purchased from Biosesang (Seoul, Republic of Korea) and EZ-CyTox assay kit from Daelillab service Co. (Republic of Korea).

### 2.2. Preparation of GSC

 GSC was grown as previously described [10]. An authenticated voucher specimen of CM (Kucari 0903) as deposited in the Herbarium at the College of Bioscience and Biotechnology, Konkuk University (Seoul, Republic of Korea). Briefly, the CM mycelium (Kucari 0906) was inoculated on germinated soybeans (*Glycine max* (L.) Merr), and cultured at 20–25°C for 4 weeks. The powdered material (1 kg) was extracted under reflux with 80% MeOH (methanol extract of GSC (GSCM)) for 48 h. The total extract (178 g, yield (w/w), 17.8%) was dissolved with water. After removing the insoluble solid particles by filtration, the liquid phase was extracted sequentially by solvents with increasing polarity (hexane, EtOAc, BuOH, and water; 1 : 10 (w/v) for all solvents) to yield 4 fractions. The liquid-liquid phase extraction was performed in Erlenmeyer flasks by shaking, and the extracts were concentrated to dryness by a rotary evaporator. Thus, we obtained the following fractions: hexane fraction (16 g, yield (w/w) 1.6%), EtOAc fraction (4.5 g, yield (w/w) 0.45%), BuOH fraction (8.25 g, yield (w/w) 0.825%), and water fraction (10.86 g, yield (w/w) 1.086%).

### 2.3. Cell Culture

The HT-29 cells (human colon cancer cells) were purchased from the American Type Culture Collection (ATCC, Manassas, VA, USA). Cells were cultured in RPMI 1640 medium (Gibco, Grand Island, NY, USA) supplemented with 10% heat-inactivated FBS (Gibco, Grand Island, NY, USA), 100 U/mL of penicillin, and 100 *μ*g/mL streptomycin at 37°C in a humidified incubator with 5% CO_2_.

### 2.4. Cell Proliferation Assay

The effect of GSC, GS, and CM BuOH extract on HT-29 cell proliferation was measured using the EZ-CyTox kit (Daelillab service Co., Republic of Korea). The assay was performed as per the manufacturer's protocol. HT-29 colon cancer cells (1 × 10^4^ cells/well) were placed in a 96-well plate and incubated with various concentrations (0, 25, 50, 75, 100, and 250 *μ*g/mL) of GSC BuOH, GS BuOH, and CM BuOH extracts for 48 h and 72 h, respectively. A fixed amount (10 *μ*L) of EZ-CyTox reagent was added to each well and incubated for an additional 1-2 h at 37°C. Cell proliferation levels were detected at an optical density (OD) of 450 nm by using an ELISA Multidetection Reader (Tecan, Mannedorf, Switzerland).

### 2.5. Morphological Analysis

HT-29 cells were placed in 6-well plates at a density of 1 × 10^6^ cells/mL. After 24 h incubation, the cells were treated with different concentrations of GSC BuOH extract. After 48 h, the cells were fixed with 3% formaldehyde and then observed under a phase-light microscope (Olympus, Tokyo, Japan) and photographed at a magnification of 100x to detect any morphological changes.

### 2.6. Cell Cycle Analysis

HT-29 cells (1 × 10^6^ cells/mL) were incubated in 6-well plates in the presence or absence of GSC BuOH extract for 48 h, after which they were harvested by trypsinization, washed twice with phosphate buffered saline (PBS), and fixed with 70% ice-cold ethanol. After centrifugation, the fixed cells were incubated with a staining solution containing 0.2% NP-40, RNase A (30 *μ*g/mL), and PI (50 *μ*g/mL) (Sigma, St. Louis, USA) in a phosphate-citrate buffer (pH 7.2). Cellular DNA content was analyzed by flow cytometry using a Becton Dickinson laser-based flow cytometer (Becton Dickinson, New Jersey, USA). At least 10,000 cells were used for each analysis, and the results were displayed as histograms. The average percentage of cells in each phase of the cell cycle was determined over 3 independent experiments.

### 2.7. Western Blotting Analysis

The HT-29 cells were treated with GSC BuOH extracts at concentrations of 0, 25, 50, 75, 100, and 250 *μ*g/mL for 48 h. Cells were lysed in the lysis buffer and the protein concentration was determined using a protein assay kit (Thermoscientific, Rockford, USA) with bovine serum albumin as the standard. Samples with equal amounts of protein were analyzed by 12% SDS-PAGE. The proteins were transferred to a polyvinylidene fluoride (PVDF) membrane using a transfer buffer. The membranes were incubated overnight at 4°C with a blocking buffer (1× PBS-T and 5% skim milk) and then incubated with antibodies against Cdc25c, cyclin B1, Bcl2, caspase-9 (1 : 2,000), and *β*-actin (1 : 3,000) for 2-3 h at room temperature with constant shaking. The membranes were washed three times in a 1× PBS-T buffer and incubated with HRP-conjugated secondary antibodies (1 : 5,000) for 1-2 h. The membranes were washed and detection of the immunoreactive bands was performed using the enhanced chemiluminescence western blotting detection system (Biosesang, Seoul, Republic of Korea).

### 2.8. Statistical Analysis

Values are presented as percentage ± SD of control. Student's *t*-test or one-way ANOVA/Dunnett's *t*-test was used to analyze the statistical significance between the CM-treated and control groups. Statistical analysis was performed using SPSS, version 12 (SPSS Inc., Chicago, IL, USA). 

## 3. Results

### 3.1. GSC Treatment Inhibited HT-29 Colon Cancer Cell Proliferation and Induced Changes in HT-29 Cell Morphology

**Figure 1:**
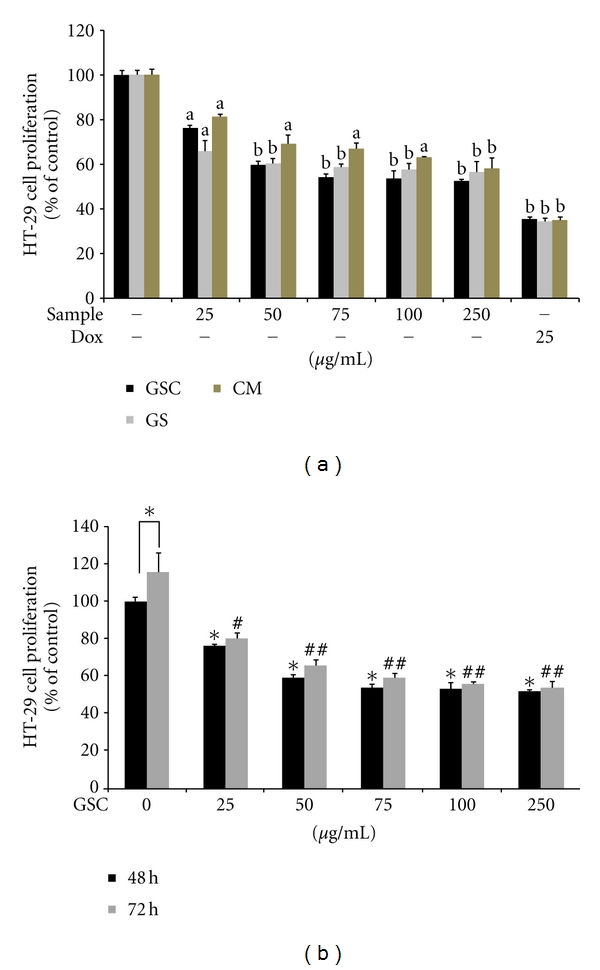
Anti-proliferative effects of GSC, GS, and CM BuOH extract on HT-29 cells. (a) HT-29 cells (1 × 10^4^/well) were treated with various concentrations (0, 25, 50, 75, 100, and 250 *μ*g/mL) of GSC, GS, CM BuOH, extract and 25 *μ*g/mL of doxorubicin for 48 h. Doxorubicin, a potent anticancer agent, was used as the positive control. The HT-29 cell proliferation was determined by the EZ-CyTox assay. One-way ANOVA followed by Dunnett's *t*-test was used for comparisons of multiple group means (^a^ *P* < 0.001 or ^b^ *P* < 0.0005 versus blank). (b) Effect of GSC BuOH extract on HT-29 cell proliferation. Time-dependent inhibition of HT-29 cell proliferation by the GSC BuOH extracts. The growth inhibition was calculated as a percentage of inhibition and compared with the values for the control. One-way ANOVA followed by Dunnett's *t*-test was used for comparisons of multiple group means (*P < 0.001 versus control (48 h), ^#^ *P* < 0.001 or ^##^ *P* < 0.0005 versus control (72 h), *P < 0.01 control (48 h) versus control (72 h)). GSC, *Cordyceps militaris* grown on germinated soybean; GS, germinated soybean; CM, *Cordyceps militaris. *

In comparison with the GS and CM BuOH extracts, GSC BuOH extract showed a significant inhibitory effect on the growth of HT-29 cells (Figure 1(a)). After a 48 h treatment with 100 *μ*g/mL of GSC BuOH, GS BuOH, and CM BuOH extracts, the proliferation of HT-29 cells reduced by 46.56% ± 3.55%, 42.56% ± 2.99%, and 36.23% ± 0.46%, respectively. Therefore, further experiments were conducted using the GSC BuOH extract. As shown in Figure 1(b), GSC BuOH extracts significantly inhibited HT-29 cell proliferation in a concentration- and time-dependent manner. However, GSC BuOH extracts did not affect cell viability of mouse macrophage RAW264.7 (data not shown).

**Figure 2:**
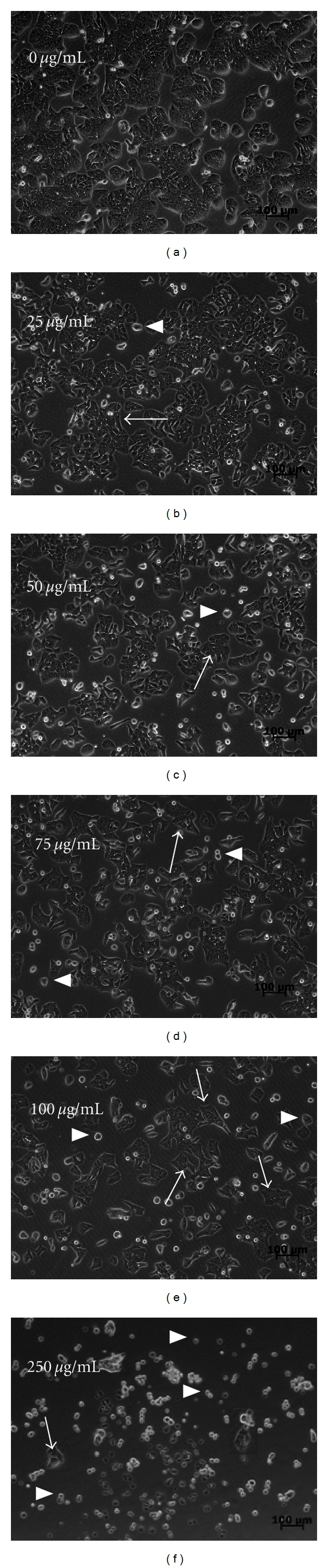
Effect of GSC BuOH extract on morphological changes in HT-29 colon cancer cells. HT-29 cells were treated with the following concentrations of GSC BuOH for 48 h. (a) 0 *μ*g/mL, (b) 25 *μ*g/mL, (c) 50 *μ*g/mL, (d) 75 *μ*g/mL, (e) 100 *μ*g/mL, and (f) 250 *μ*g/mL of GSC BuOH extract. The cell morphology was photographed by EVOS inverted microscope at 100x (Advanced Microscopy Group, Mill Creek, USA). The arrow indicates cells with irregular size and shape. The arrowhead indicates single cells and loss of colony formation (bar = 100 *μ*m). GSC, *Cordyceps militaris* grown on germinated soybean.

We also found morphological changes in HT-29 cells after GSC BuOH extract treatment (Figure 2). After treatment with GSC BuOH (0, 25, 50, 75, 100, and 250 *μ*g/mL) for 48 h, the cells showed morphological features such as loss of colony formation and irregular size and shape, which were not found in the cells of the control group.

### 3.2. GSC Induced G2/M Phase Cell Cycle Arrest in HT-29 Cells

**Figure 3:**
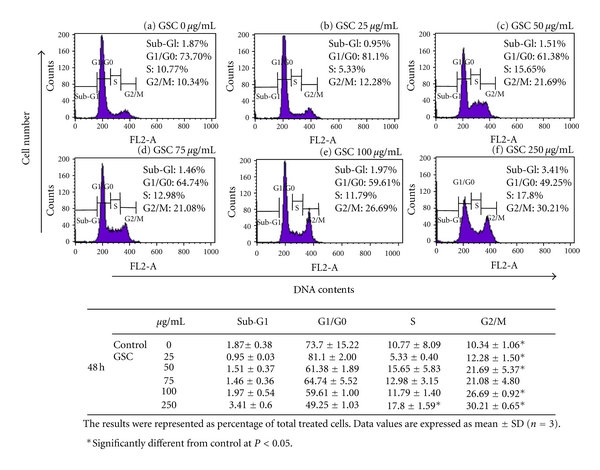
GSC BuOH extract induced G2/M phase cell cycle arrest in HT-29 cells. Cell cycle progression was examined by flow cytometry. (a) 0 *μ*g/mL, (b) 25 *μ*g/mL, (c) 50 *μ*g/mL, (d) 75 *μ*g/mL, (e) 100 *μ*g/mL, and (f) 250 *μ*g/mL. One representative of 3 independent experiments is shown (48 h treatments). Histograms show the findings during sub-G1, G1/G0, S, and G2/M phases of HT-29 cells. One-way ANOVA followed by Dunnett's *t*-test was used for comparisons of multiple group means (*P < 0.05 or *P < 0.001 versus control).

Uncontrolled cell proliferation is a characteristic of cancer cells [16]. To prove the inhibitory mechanism of GSC BuOH extract against colon cancer cells, we examined the cell cycle alteration of HT-29 cells by flow cytometry. After GSC BuOH extract treatment for 48 h, the DNA contents of the G2/M phase of HT-29 cells increased in a concentration-dependent manner than those of the control group. The percentage of cells in G2/M phase were 12.28% ± 1.50%, 21.69% ± 5.37%, 21.08% ± 4.80%, 26.69% ± 0.92%, and 30.21% ± 0.65% compared to that in control (10.34% ± 1.06%), when treated with 25, 50, 75, 100, and 250 *μ*g/mL of GSC BuOH extract for 48 h, respectively (Figure 3). These results indicate that GSC BuOH extract caused G2/M cell cycle arrest in HT-29 cells.

### 3.3. GSC Reduced the Protein Levels of Cyclin B1 and Cdc25c in HT-29 Cells

**Figure 4:**
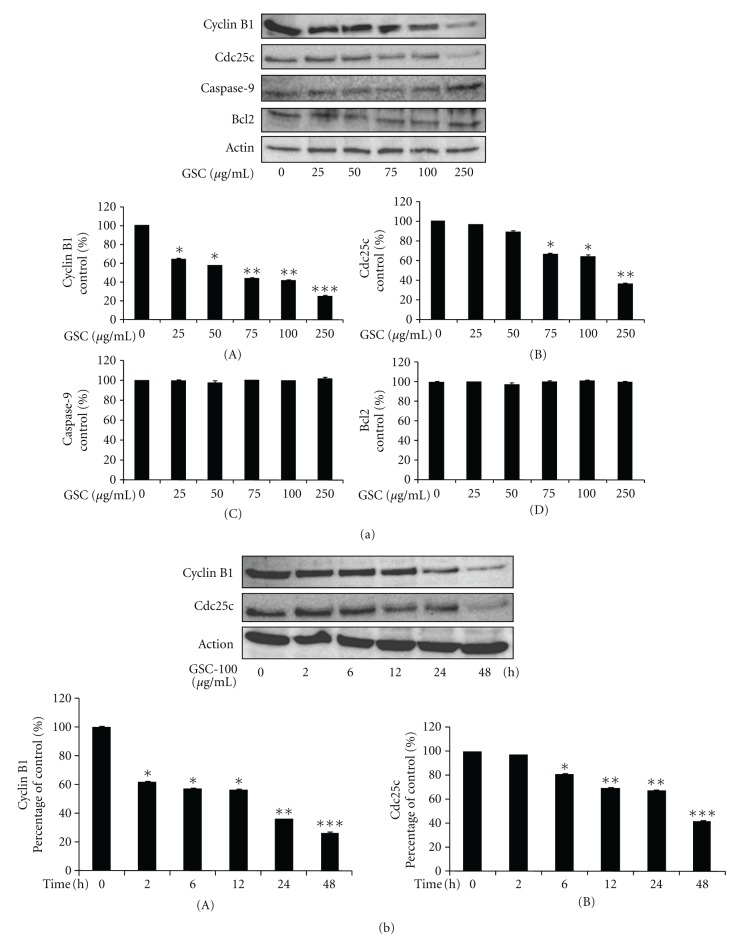
Effect of GSC BuOH extract on the protein expression levels of cyclin B1 and Cdc25c in HT-29 cells. (a) HT-29 cells were treated with the indicated concentrations (0, 25, 50, 75, 100, and 250 *μ*g/mL) of GSC BuOH extract for 48 h. Cell lysates were processed for western blot analysis with anti-cyclin B1, anti-Cdc25c, anti-Bcl2, anticaspase-9, and anti-*β*-actin antibodies. Densitometric ((A)–(D)) analysis of 3 independent western blots (mean ± SD) expressed in terms of a percentage of the values for the control groups (*P < 0.05; **P < 0.01; ***P < 0.005). (b) HT-29 cells were treated with 100 *μ*g/mL of GSC BuOH extract for 2, 6, 12, 24, and 48 h. Cell lysates were processed for western blot analysis with anti-cyclin B1, anti-Cdc25c, and anti-*β*-actin antibodies. *β*-Actin was used as an internal control. Figures are representative of 3 independent experiments. Densitometric analysis of the bands of three western blots (mean ± SD) expressed in terms of a percentage of the values for the control groups (*P < 0.05; **P < 0.01; ***P < 0.005).

To determine the molecular mechanisms underlying the G2/M arrest by GSC BuOH extract, the levels of the molecules involved in the G2/M phase of the cell cycle were checked. GSC BuOH extract-treated cells strongly blocked the expression of cyclin B1 and Cdc25c protein in HT-29 cells in a concentration-dependent (0, 25, 50, 75, 100, and 250 *μ*g/mL) and time-dependent (0, 2, 6, 12, 24, and 48 h) manner (Figures 4(a) and 4(b)). However, the GSC BuOH extract had no effect on the levels of apoptosis-related proteins like Bcl2 and caspase-9 (Figure 4(a)).

## 4. Discussion

Due to the increasing incidences and relatively low remission rates of colon cancers, there is a need to establish more effective treatment regimens by adopting novel and innovative approaches [16]. The use of active medicinal compounds or extracts from traditional medicines or natural sources is considered as one such alternative treatment approach. Many naturally derived compounds/extracts are considered safe as they are obtained from commonly consumed foodstuffs. CM is a well-known medicinal mushroom that has been used in oriental medicine for treating various diseases, including cancers. Previous studies have demonstrated that CM has a wide range of pharmacological activities, including immunomodulatory and anti-inflammatory activities [7–11].

Cancer cells generally exhibit few characteristics such as high proliferation, migration, and matrix-invasion potentials [17, 18]. Inhibition of tumor growth is one of the therapeutic targets in the development of anticancer agents. The regulation of tumor cell growth and the induction of cell death are the 2 major ways to inhibit tumor growth [19]. The present study evaluated whether GSC BuOH extracts had anti-proliferative activities against HT-29 cells. Although GSC BuOH, GS, and CM BuOH extracts exhibited anti-proliferative activity against HT-29 cells, GSC BuOH extracts showed anti-proliferative activity with the lowest IC_50_ (100 *μ*g/mL) value. Severely distorted HT-29 cells and loss of colony formation ability were observed after GSC BuOH treatment. We analyzed the changes in HT-29 cell cycle progression after GSC BuOH treatment by flow cytometry. Cellular proliferation is controlled by various genetically defined checkpoints, which ensure the progression of cells through the various stages of the cell cycle [20]. In cancer cells, cell cycle checkpoint control systems are known to be disrupted through the accumulation of mutations [21]. In the G2/M phase, damaged cells have the opportunity to repair DNA or permanently arrest cell growth if the degree of damage is severe [22]. Several anticancer agents arrest the cell cycle in G2/M phase, and then induce apoptosis and necrosis, resulting in cell death [23, 24]. In general, the G2/M transition is regulated by a complex of cell-division cyclins, namely, Cdc2 and a B-type cyclin [22]. The protein tyrosine phosphatase, Cdc25c, plays the role of a mitotic activator by dephosphorylating Cdc2/p34, which forms the Cdc2/cyclin B1 complexes that permit cells to enter into mitosis [25]. Many studies showed that Cdc2/p34 kinase activity was enhanced in some human cancer cells because of their genetic and epigenetic alterations [22]. Therefore, we investigated whether the expressions of the molecules involved in G2/M transition in HT-29 cells were altered after GSC BuOH extract treatment. We found that GSC treatment resulted in downregulation of Cdc25c and cyclin B1 expression in HT-29 colon cancer cells. The results of the present study indicated that GSC caused G2/M-phase cell cycle arrest along with a decrease in the levels of cyclin B1 and Cdc25c, which are involved in cell cycle progression from the G2/M phase. In addition, the identification of such compounds will improve our understanding of the anti-proliferative activities of GSC. Further experiments need to be done to clarify the anti-proliferative mechanisms of these identified compounds.

## 5. Conclusion

In conclusion, GSC BuOH extracts might act as an effective anti-proliferative agent by inducing G2/M cell cycle arrest in colon cancer cells.
